# CpG Methylation Profiles of HIV-1 Proviral DNA in Individuals on ART

**DOI:** 10.3390/v13050799

**Published:** 2021-04-29

**Authors:** Valerie F. Boltz, Cristina Ceriani, Jason W. Rausch, Wei Shao, Michael J. Bale, Brandon F. Keele, Rebecca Hoh, Jeffrey M. Milush, Steve G. Deeks, Frank Maldarelli, Mary F. Kearney, John M. Coffin

**Affiliations:** 1 HIV Dynamics and Replication Program, Center for Cancer Research, NCI-Frederick, Frederick, MD 21702, USA; cristina_ceriani@med.unc.edu (C.C.); rauschj@mail.nih.gov (J.W.R.); mib4004@med.cornell.edu (M.J.B.); fmalli@mail.nih.gov (F.M.); kearneym@mail.nih.gov (M.F.K.); 2 University of North Carolina HIV Cure Center, University of North Carolina at Chapel Hill, Chapel Hill, NC 27599, USA; 3 Advanced Biomedical Computing Center, Frederick National Laboratory for Cancer Research, Frederick, MD 21702, USA; shaow@mail.nih.gov; 4 Weill Cornell Graduate School of Medical Sciences, Cornell University, New York, NY 10065, USA; 5 AIDS and Cancer Virus Program, Frederick National Laboratory for Cancer Research, Frederick, MD 21702, USA; keelebf@mail.nih.gov; 6 Department of Medicine, University of California, San Francisco, CA 94143, USA; Rebecca.Hoh@ucsf.edu (R.H.); jeff.milush@gmail.com (J.M.M.); Steven.Deeks@ucsf.edu (S.G.D.); 7 Department of Molecular Biology and Microbiology, Tufts University, Boston, MA 02129, USA; john.coffin@tufts.edu

**Keywords:** CpG dinucleotide methylation, HIV latency, single genome sequencing, RNA expression, transcriptional silencing

## Abstract

The latent HIV-1 reservoir is comprised of stably integrated and intact proviruses with limited to no viral transcription. It has been proposed that latent infection may be maintained by methylation of pro-viral DNA. Here, for the first time, we investigate the cytosine methylation of a replication competent provirus (AMBI-1) found in a T cell clone in a donor on antiretroviral therapy (ART). Methylation profiles of the AMBI-1 provirus were compared to other proviruses in the same donor and in samples from three other individuals on ART, including proviruses isolated from lymph node mononuclear cells (LNMCs) and peripheral blood mononuclear cells (PBMCs). We also evaluated the apparent methylation of cytosines outside of CpG (i.e., CpH) motifs. We found no evidence for methylation in AMBI-1 or any other provirus tested within the 5′ LTR promoter. In contrast, CpG methylation was observed in the *env-tat-rev* overlapping reading frame. In addition, we found evidence for differential provirus methylation in cells isolated from LNMCs vs. PBMCs in some individuals, possibly from the expansion of infected cell clones. Finally, we determined that apparent low-level methylation of CpH cytosines is consistent with occasional bisulfite reaction failures. In conclusion, our data do not support the proposition that latent HIV infection is associated with methylation of the HIV 5′ LTR promoter.

## 1. Introduction

Latent HIV-1 infection of CD4+ T cells is established in acute infection [1,2] and maintained by homeostatic and antigen-driven T-cell proliferation throughout the lifetime of the individual [3]. Infection persists even in individuals whose viremia is controlled by ART, creating a major barrier to a cure [4,5,6].

Transcriptional silencing associated with HIV-1 latency has been proposed to be linked to cytosine methylation in CpG dinucleotide clusters in the promoter region of the pro-viral 5′ LTR [7,8]. Indeed, promotor methylation has been shown to regulate human gene expression [9,10], regulate expression of other retroviruses, such as human T cell leukemia virus [11] and murine leukemia virus [12], and regulate expression of endogenous retroviruses [13]. Prior evidence linking provirus methylation to the maintenance of HIV-1 latency has been mixed. Some studies have reported high levels of CpG methylation in the 5′ LTR promotor region, suggesting an association with HIV latency [7,14,15], while others have found this region to be unmethylated, and concluded that the latent viral reservoir must be maintained by another mechanism [16,17]. However, none of these prior studies have linked a specific provirus residing in the latent reservoir and capable of producing an infectious virus to its methylation state.

**Figure 1:**
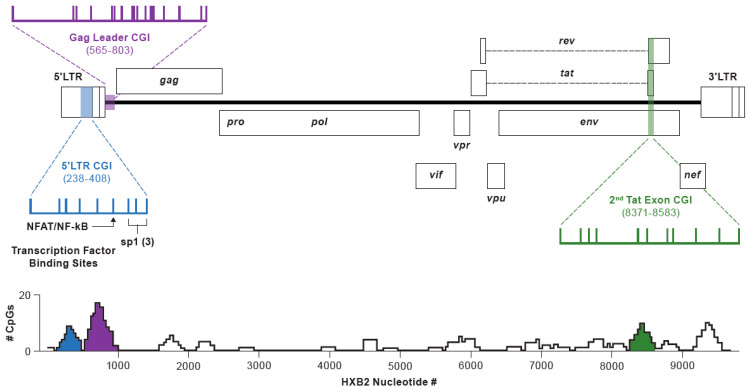
Structure of the HIV-1 genome illustrating the three canonical CGIs in the HXB2 genome, CpG positions within each, and transcription factor binding and initiation sites. CpG density vs. HXB2 bp numbering (GenBank: K03455.1) in a 201 bp sliding window (i.e., reference +/− 100 bp in the 5′ and 3′ directions). Methylation status of the CGIs evaluated in this study are shaded to match the color coding.

The discovery and characterization of a T cell clone harboring a replication-competent HIV-1 provirus called AMBI-1 [18] has provided a unique opportunity to overcome this limitation. The full-length AMBI-1 sequence, including its host site of integration, was first described by Simonetti et al. [19]. Constituting approximately 8–10% of the total provirus population and persisting for >4 years, the AMBI-1 clone made repeated sampling for the determination of 5′ LTR methylation feasible. Moreover, only 2.3% of AMBI-1 infected cells in PBMCs expressed unspliced cell-associated HIV-1 RNA during ART, and those were only at low levels (≤10 copies/cell) [20], indicating that more than 97% of the proviruses in the clonal population were transcriptionally silent and thus representative of the latent HIV-1 reservoir. Other clonally expanded intact proviruses in individuals on ART have also been identified [21,22,23]. Equipped with this knowledge, we investigated the methylation state of the CpG islands (CGI) in the 5′ LTR, *gag*-leader, and second *tat* exon (Figure 1) at the single pro-viral level of the AMBI-1 clone and other proviruses obtained from PBMCs and LNMCs from the same individual. PBMCs and LNMCs from three other individuals with similarly low levels of intracellular unspliced HIV-1 RNA during ART were also evaluated [23,24].

We found no evidence for CpG methylation above the assay background in the LTR or *gag*-leader CG island in the AMBI-1 provirus or any other provirus sampled from Patient 1 or any other study participant. By contrast, there was detectable CpG methylation in the *tat* exon 2 CG island in both PBMCs and LNMCs, a region not known to be associated with pro-viral expression. Thus, despite the precedent from other retroviral systems, we did not find evidence for involvement of CpG DNA methylation of the 5′ LTR in transcriptional silencing of latent HIV.

## 2. Methods

### 2.1. Donor Samples

At the time of initial HIV-1 diagnosis, Patient 1 was participant AVBIO2-02 enrolled in NIH protocol 08-I-0221, conducted at the NIH Clinical Center in Bethesda, MD, USA. Combination ART was initiated. An average of 5 × 10^6^ to 1 × 10^7^ total PBMCs were obtained 11 years after initiating suppressive ART and used for this study (Supplemental Table S1). The lymph node sample was taken postmortem, 13 years from the initial diagnosis, and an average of 5 × 10^6^ to 1 × 10^7^ total LNMCs were also used for this study. Patient 1 reported complete ART adherence until his death. His last documented viral load of 107 occurred five months before his expiration date. HIV sequences from this low-level persistent plasma viremia from Patient 1 contained no drug resistance mutations and was consistent with expansion of the AMBI-1 clone throughout the patient’s history [19]. PBMCs and LNMCs were used for our single-genome sequencing (SGS) assay in conjunction with bisulfite-mediated C to U conversion. Three other donors from the San Francisco SCOPE cohort (NCT00187512), PIDs 1079, 1683, and 2669, all of whom had chronic HIV-1 infection that was stably suppressed by combination ART, were also included in the study (5 × 10^6^ to 1 × 10^7^ PBMCs and LNMCs were analyzed) [23,24] (Supplemental Table S1). Studies were approved by the Institutional Review Boards of the University of California San Francisco and the National Institute of Allergy and Infectious Diseases. All participants provided written informed consent to participate in the study.

### 2.2. Single Genome Sequencing of Non-Bisulfite Treated Genomic DNA

To characterize sequence variation among CGIs in donors, parallel samples of HIV-infected human genomic DNA (gDNA) purified from 1 × 10^6^ PBMCs or 1 × 10^6^ LNMCs were subjected to SGS as previously described [25,26], except with modifications to the primers, which are described in Supplemental Table S2a. Sequences from at least 20 individual proviruses were obtained from each donor, cell type, and region (5′ LTR, *gag*-leader and the second *tat* exon), and were used to construct consensus alignments to define consensus CpG sites for each respective data set. Also defined within the consensus alignments were the CpH sites (cytosines not in CpG sites). Consensus alignments for each donor were used as references for sequences obtained in bisulfite methylation detection experiments.

### 2.3. Primer Design for Methylation Detection

The absence of CpG dinucleotides in the region-specific priming sites from each donor was the main criterion for selecting primers for nested PCR of bisulfite-treated DNA. The Zymo Research Bisulfite Primer Seeker program https://www.zymoresearch.com/pages/bisulfite-primer-seeker used to facilitate the converted primer design, where outer and nested primers were specific for the bisulfite-converted DNA. Using SGS as references, all converted primers were donor specific, ensuring complementarity in each donor in the region being amplified. To amplify the 5′ LTR CGI in the first PCR step, we used a reverse primer, complementary to *gag*-leader sequences for the bisulfite-converted DNA, thus ensuring that the 3′ LTR sequences would not be amplified. In addition, primers that were complementary to the bisulfite-converted DNA were designed to specifically amplify the AMBI-1 clone. Since host DNA sequences flanking the AMBI-1 provirus integration site are known [19], two outer, forward primers specific for bisulfite-converted DNA for 5′-LTR amplification were designed, one specific for the host sequence flanking the AMBI-1 5′ LTR junction and the other to a sequence in U3 upstream from the HIV-1 transcription start site (Supplemental Table S2b). These primers, in combination with a reverse primer complementary to the *gag*-leader just downstream of U5, were used to amplify the converted AMBI-1 5′ LTR specifically or the converted 5′ LTR sequences in general from Patient 1, respectively. Sequences of all converted primers used in the study are reported in Supplemental Table S2b–d.

### 2.4. Methylation Site Detection and Analysis

The EpitectR Bisulfite Kit (Qiagen cat# 59104) was used to selectively convert unmethylated Cs to Us in gDNA samples. For each successive experiment, gDNA purified from 10^6^ donor PBMCs or LNMCs was dispensed into ten 500 ng aliquots and incubated with 85 µL of dissolved bisulfite mix for 5 h in a thermocycler, alternating between three cycles of denaturation at 95 °C and incubation at 60 °C according to the manufacturer’s instructions. Once the cycling was complete, the bisulfite-converted gDNA was purified and SGS was performed using donor-specific converted primers for the bisulfite-treated gDNA until at least 20 individual converted SGS were obtained as described for non-bisulfited treated gDNA. SGS from untreated and bisulfited-treated assays were aligned to HXB2 and trimmed to the same starting position in each region. Converted DNA sequences were compared to their respective consensus alignments. Using Quma (http://quma.cdb.riken.jp), an online quantification tool for methylation analysis, the percent methylation was determined by dividing the number of methylated CpG sites by the total number of true CpG sites. The denominator of total CpG sites in the bisulfite-converted sequences was always verified to include only those that contain a CpG or dUpG dinucleotide (appearing as TpG in the converted sequence). The percentages of unconverted Cs in CpH sites were similarly determined and used to identify the reaction failure background.

### 2.5. Controls for Bisulfite Treatment

To address the issue of bisulfite reaction failures and incomplete conversion, i.e., when unmethylated Cs are not converted to Us and rare instances in which 5mC-to-U conversion does occur, negative and positive control templates were generated. In brief, two unmethylated, negative control templates for the 5′ LTR region of pNL4-3 were generated, first by PCR amplification and second by transformation and harvesting of the plasmid from a methyltransferase deficient (Dam-/Dcm-) strain of *E. coli* (NEB; #C2925I). To generate the methylated, positive control template, 1 × 10^6^ CEM cells were spiked with approximately 50 ACH2 cells, each containing approximately one stably integrated HIV-1LAV provirus (NIH AIDS Reagent Program catalog #349) [27]. Following the manufacturer’s instructions, aliquots of the gDNA mixtures were treated with CpG methyltransferase (M.Sssl; NEB: M0226M), an enzyme that efficiently converts C to 5mC in CpG motifs in double stranded DNA. All control templates were processed exactly as described for methylation site detection and analysis.

### 2.6. Statistics

For each dataset, the numbers of unconverted cytosines and total cytosines at CpH and CpG sites, respectively, were compared in 2 × 2 contingency tables and two-tailed Fisher’s exact tests and chi-squared with Yates correction tests conducted to determine whether cytosine methylation levels relative to the background or to other samples were statistically significant.

## 3. Results

### 3.1. Study Design

Three regions in HIV-1 proviruses were enriched for CpG dinucleotides, as evidenced from sequence analysis of CpG density in a 200-nucleotide sliding window (Figure 1). Levels of DNA methylation within these three canonical CpG islands in the HIV-1 genome were examined in this study. These encompass (i) the U3 region of the LTR extending through the transcription factor binding sites in the 5′ LTR (HXB2 reference positions 238–408), (ii) a segment initiating within the *gag*-leader region and extending just into the p17 MA region at the 5′ end of *gag* (595–803), and (iii) a region within the second exon of *tat* in which the *env, tat*, and *rev* reading frames overlap (8371–8583). The size and positioning of these elements relative to the full HIV-1 genome are depicted in Figure 1. In addition, Figure 1 shows nine possible CpG sites within the 5′ LTR CG island, 17 within the *gag*-leader region, and 11 within the second exon of *tat*. Note that the distribution of CpG dinucleotides in these CGIs vary among participants, as HXB2 was used as a reference, and not all of the indicated sites are present in all donors sampled.

### 3.2. CpH Methylation and Assay Validation

Low-level physiological methylation at C residues followed by A, C, or T (referred to hereafter as CpH sites) has been reported [28]. It was possible, however, that the observations of bisulfite non-conversion of CpH to dUpH could have been due to a low level of incomplete conversion of unmethylated cytosines rather than methylation. To determine the frequency of CpH-to-dUpH non-conversion at CpH sites in our bisulfite-treated samples, we measured cytosine non-conversion in negative control experiments using templates that could not possibly contain 5mC. In all of the experiments presented here, C-to-U non-conversion at CpH sites in the treated samples was used to calculate bisulfite reaction failure rates, which were in turn used in all subsequent studies to estimate the contribution of this assay background to non-conversion at CpG sites in the same sequence segments.

**Figure 2:**
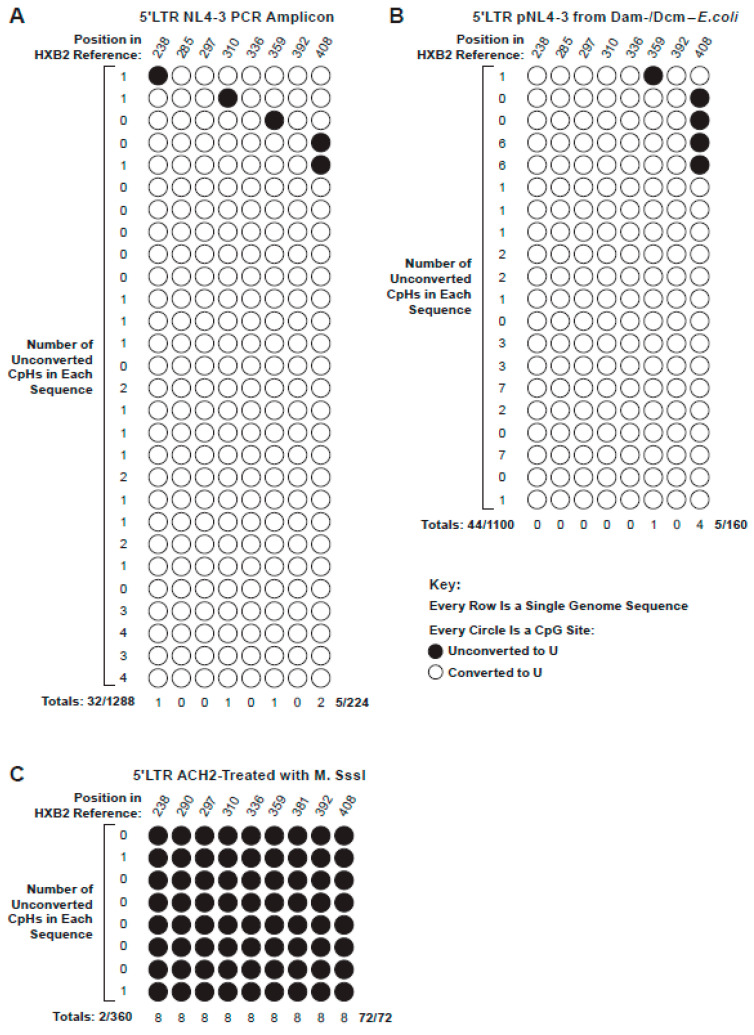
Bisulfite conversion pattern of negative and positive control DNAs SG sequences of the 5′LTR. Each row represents a single provirus with nine CpG sites. A white circle indicates that the cytosine in the CpG dinucleotide was converted to dU. A black circle indicates that the cytosine was not converted. The column of numbers on the left shows the number of non-converted C residues at sites other than CpG (i.e., CpH sites). (**A**) Twenty-eight treated SG sequences of nonmethylated HIV-1 NL4-3 PCR product. (**B**) Twenty SG sequences of DNA from plasmid pNL4-3 grown in Dam-/Dcm- chemically competent *E coli*. (**C**) Eight SG sequences of pro-viral HIV-1 DNA from CEM/ACH2-M.SssI cells. The frequencies of conversion at the CpG and CpH sites in A and B were not significantly different (*p* = 0.99 and 0.83).

For this purpose, we first prepared negative control templates consisting of 5′ LTR amplicons of HIV-1 NL4-3 DNA produced in PCR reactions containing standard, non-methylated dNTPs. Bisulfite treatment of these amplicons and subsequent SGS produced 28 sequences, each of which was assessed for cytosine non-conversion. Collectively, non-conversion of unmethylated cytosines within CpG sites was 2.2%, i.e., five of 224 cytosines in CpG sites were not converted to uridine in the bisulfite reaction (Figure 2A). Similarly, only 32 of the 1288 CpH sites were not converted (2.5%), a rate not significantly different from that observed for CpG dinucleotides (*p* = 0.99) (Supplemental Table S3). A second negative control experiment was repeated using purified plasmid DNA for bisulfite treatment harvested from a methyltransferase deficient (Dam-/Dcm-) strain of *E. coli*. The backgrounds of unconverted, nonmethylated Cs in CpG and CpH sites across the 20 plasmid-derived SG sequences analyzed were 3.1% (5/160) and 4% (44/1100), respectively (Figure 2B), and the difference between these non-conversion rates was again not found to be statistically significant (*p* = 0.83) (Supplemental Table S3).

Positive control templates were also generated to measure reaction selectivity (i.e., the conversion rate of methylated cytosine). This experiment involved treating genomic DNA from mixtures of CEM and latently infected ACH-2 cells containing one integrated pro-viral copy of LAV with CpG methyltransferase, an enzyme that efficiently methylates cytosines within CpG motifs. Subjecting these templates to bisulfite treatment and SGS yielded eight 5′ LTR sequences, in which 100% of the CpG sites were not converted (72/72) (Figure 2C), indicative of a very high selectivity and specificity for this assay. The similar rates of conversion failure of the CpG and CpH sites using these model substrates allowed us to use CpH non-conversion as an internal control for the assay background in all subsequent experiments.

### 3.3. Methylation Status of the 5′LTR Promoter in the HIV AMBI-1 Clone

**Figure 3:**
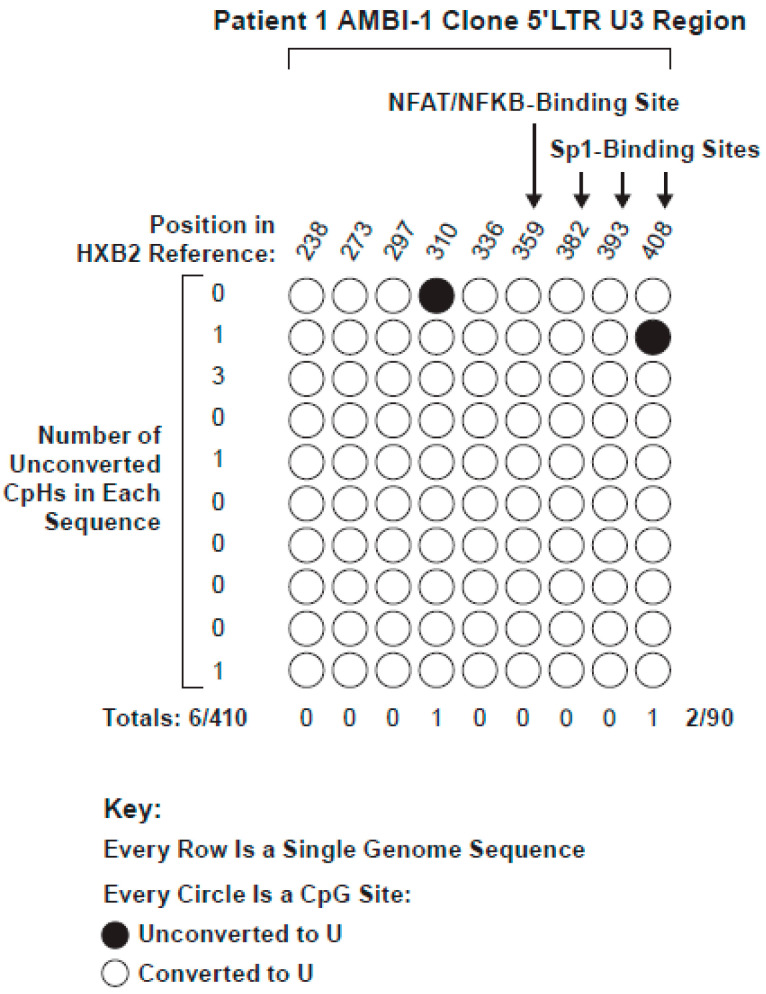
Level of conversion of the 5′ LTR CG island from 10 pro-viral AMBI-1 clone SG sequences from Patient 1 after bisulfite treatment of gDNA from PBMCs and LNMCs. The frequency of non-conversion of the CpG sites (2.2%) was not significantly different from that of the CpH sites (1.5%), shown to the left of each row (*p* = 0.64).

The CpG island in the LTR of the AMBI-1 clone contained nine CpG sites. Its 5′LTR was selectively amplified from 20 bisulfite reactions performed on 500 ng DNA aliquots obtained from Patient 1 PBMCs and 30 bisulfite reactions from Patient 1 LNMCs using bisulfite-converted, AMBI-specific primers. We obtained 10 bisulfite-converted AMBI-1 clone 5′ LTR single genome sequences (Figure 3). Of the nine possible CpG sites across each of the 10 samples (90 sites in total), only two cytosines remained unconverted, for an apparent overall methylation rate of 2.2%. Similarly, only six of the 410 CpH sites in the AMBI-1 clone LTR were not converted, yielding an overall assay background of 1.5%. The difference between the CpG and CpH apparent methylation levels was not statistically significant (*p* = 0.64) (Supplemental Table S4), indicating that the 5′ LTR promoter of the replication competent AMBI-1 provirus was consistently unmethylated, despite the absence of detectable expression by 95% of proviruses [24].

**Figure 4:**
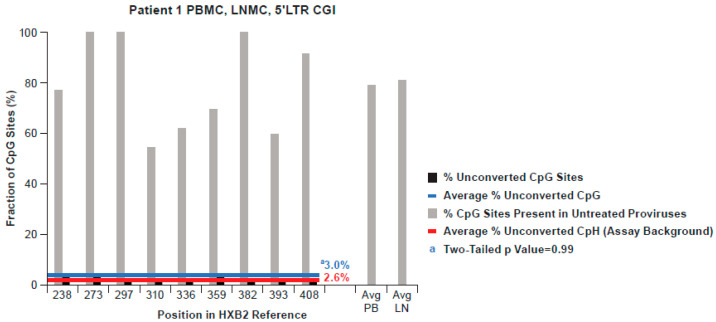
Conversion pattern of 77 bisulfite-converted 5′ LTR SG sequences from the AMBI-1 clone and from PBMC and LNMC pro-viral DNA from Patient 1. The black bars indicate the percentage of non-conversion in the population of proviruses at each of the nine CpG dinucleotide sites in the 5′ LTR, averaging 3.0% overall. The red line shows the average assay background of unconverted cytosines at non-CpG sites. The grey shading indicates the conservation of CpG sites in the population of 90 non-bisulfite treated SG sequences from pro-viral 5′ LTR DNA in Patient 1.

To determine whether the lack of detectable methylation was specific for the AMBI-1 provirus, we obtained 70 and 20 5′ LTR promoter region single genome sequences from Patient 1 PBMCs and LNMCs, respectively, which were used to build consensus CpG island sequence references. We further analyzed an additional 67 bisulfite-converted 5′ LTR sequences from other proviruses in Patient 1 PBMCs and LNMCs (presented together with the AMBI-1 clone sequences in Figure 4). Pro-viral sequences obtained from Patient 1 PBMCs and LNMCs are shown in aggregate, as all of the same CpG sites are represented and comparably distributed in the CGIs in both PBMCs and LNMCs, as shown in Figure 4. In addition, HIV-1 LTR sequences obtained from PBMCs and LNMCs intermingled on a neighbor joining tree, indicating that they were from the same population (Supplemental Figure S1A). The average apparent fractional CpG methylation across the 5′ LTR of Patient 1 was 3.0%, shown by the blue line in Figure 4 (Supplemental Table S4). The average reaction failure background of unconverted cytosines across the 5′ LTR of Patient 1 was 2.6% and is shown in Figure 4 by the red line. As in the AMBI-1 clone sequence analysis, the difference between the apparent fractional CpG methylation and assay background was not significantly different (*p* = 0.999) (Supplemental Table S4). Hence, measured non-conversion rates for CpH sites in patient samples were consistent with those in both of the negative control experiments measured, indicating that physiological CpH methylation was negligible and our experimental approach was valid.

**Figure 5:**
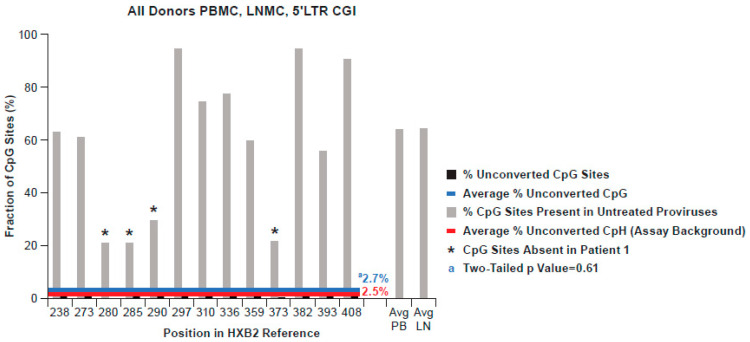
Aggregate % of non-conversion of CpG dinucleotides in the 5′ LTR of all four donors from 213 bisulfite-treated SG sequences from HIV-1 PBMC and LNMC pro-viral DNA. The average percentage of non-conversion in the 5′ LTR CpG island across the four donors was not significantly different from that of cytosines at CpH sites in all genomes averaged (*p* = 0.61).

Additionally, as shown in Figure 4, fluctuation in the fraction of CpG sites present at each position in the untreated genomes was quite evident. Specifically, only three of the nine CpG dinucleotide sites were present in 100% of the individual LTR proviruses evaluated, while six were present at frequencies ranging from 50% to 90%. In addition, four sites found in differing amounts among the other three donors were not present in any Patient 1 sequence, as illustrated in Figure 5, signifying that, while CGIs were conserved, the distribution of the CpG sites within them varied.

### 3.4. Methylation Status of the 5′LTR Promoter in All Donors

PBMC and LNMC samples from the three other donors (PIDs 1079, 1683, and 2669) were similarly processed, resulting in 50, 49, and 37 bisulfite-converted 5′ LTR single genomes, respectively, and are shown in aggregate with Patient 1 in Figure 5. The average apparent methylation of CpG dinucleotides in 5′ LTRs from the three donors was 2.8%, 3.0%, and 2.0%, respectively, fractions not significantly different from the CpH background (2.6%; 2.3%, and 2.5%; *p* = 0.4 to 0.86; Supplemental Table S4). Figure 5 also illustrates the frequencies of individual CpG sites in 5′ LTR populations across all donors (20–100%). In Figure 5, the sites depicted with an asterisk indicate sites absent in Patient 1 and variably absent in the other donors as well.

### 3.5. Methylation Status of the CpG Island in the gag-Leader

Taken together, these results do not support a role for methylation of CpG dinucleotides in the LTR in the transcriptional silencing of HIV proviruses during suppressive ART. To extend our analyses further, we investigated C methylation in the two other prominent CGIs, in the *gag*-leader and *tat* exon 2.

**Figure 6:**
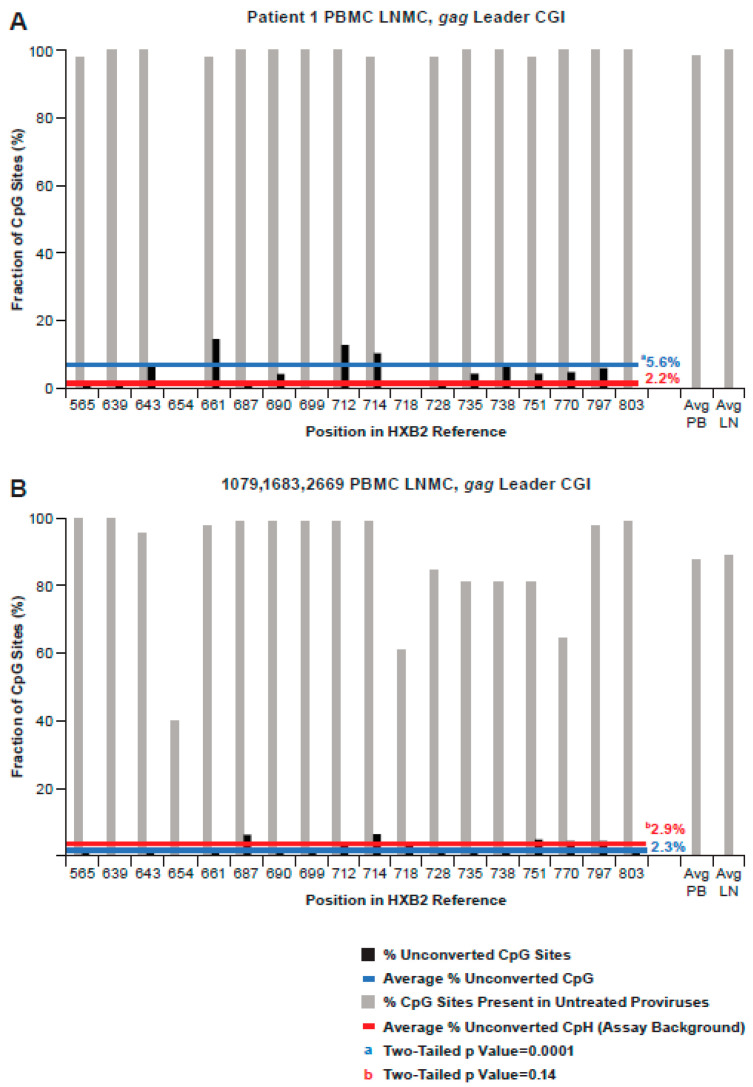
Bisulfite conversion of CpG dinucleotides in the *gag*-leader. (**A**) Conversion pattern of 47 bisulfite-treated SG sequences from HIV-1 PBMC and LNMC pro-viral DNA from Patient 1. The average percentage of non-conversion in the population of genomes at each of the 16 CpG dinucleotide sites in the *gag*-leader region was 5.6%. The average assay background of unconverted cytosines at non-CpG sites averaged 2.2% (*p* = 0.0001). (**B**) Conversion pattern of 123 bisulfite-treated SG sequences from HIV-1 PBMC and LNMC pro-viral DNA from PIDs 1079, 1683, and 2669. The average assay background of unconverted cytosines at non-CpG sites was 2.9%. The average percentage of non-conversion in the population of proviruses at each of 18 CpG dinucleotide sites in CGI was below the assay background.

Methylation of the *gag*-leader CGI was also analyzed in proviruses from PBMC and LNMC samples from all donors, resulting in 170 bisulfite-converted sequences (Figure 6). The 47 sequences obtained from Patient 1 samples are shown separately from the others (Figure 6A), as there was a significant difference in the average percent methylation across the region (5.6%) relative to the assay background (2.2%; *p* = 0.0001) (Supplemental Table S5). This difference was driven by fractional methylation levels of 15%, 13%, and 11% at CpG sites 661, 712, and 714, respectively. By contrast, only 2.3% of CpG sites in the 123 sequences from donors 1683, 1079, and 2669 were unconverted, despite the presence of CpG at the same three sites, a rate not significantly different from the background (2.9%; Figure 6B; *p* = 0.14; Supplemental Table S5). Again, however, apparent methylation at CpG site 714 in these samples, and, to a lesser extent, 687, exceeded the background levels (*p* = 0.03 and 0.06 respectively). Among the 47 sequences obtained from Patient 1, seven were potentially from the AMBI-1 clone. There was not a significant difference between unconverted CG sties (5/90) and unconverted CpH sites (5/280) (*p* = 0.07) across the region in these seven sequences, trending more toward the sequence populations from the other three donors. Collectively, our analysis showed that, like 5′ LTR CGIs, *gag*-leader CGIs were mostly unmethylated, although some sporadic CpG methylation in the *gag*-leader region was evident in one donor.

### 3.6. Methylation Status of an Intragenic CGI Encompassing the Second Exon of Tat

**Figure 7:**
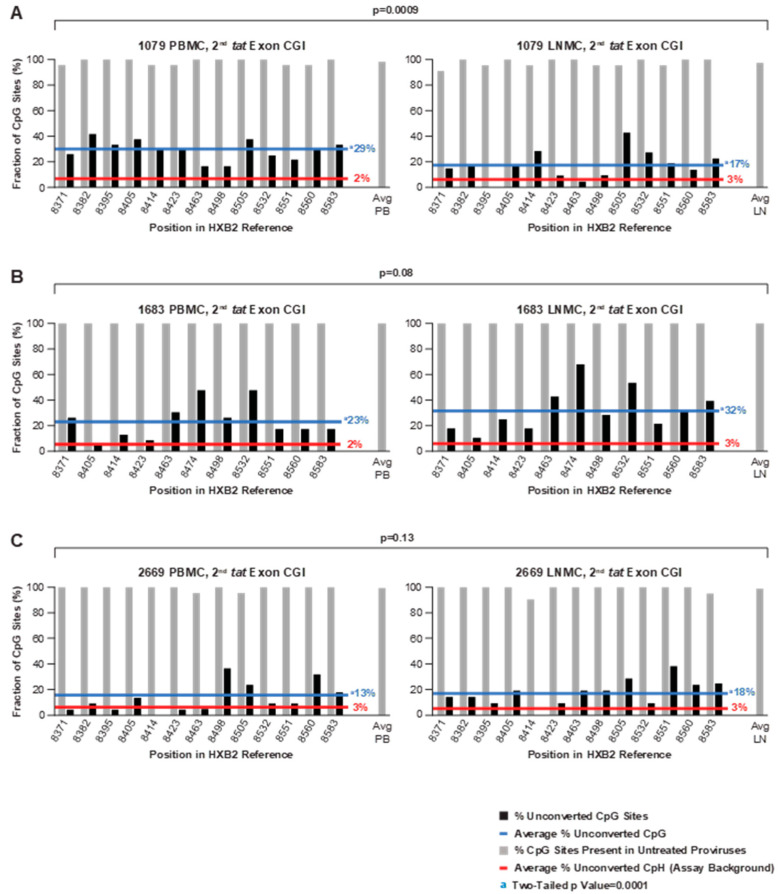
Conversion patterns of 2nd *tat* exon CGIs in proviruses from PBMCs and LNMCs from donors 1079, 1683, and 2669. (**A**) The difference between the levels of conversion between PBMCs (29%) and LNMCs (17%) was significant in PID 1079, *p* = 0.0009. (**B**,**C**) PID 1683 and PID 2669 resulted in similar levels of non-conversion between PBMCs and LNMCs, *p* = 0.08 and 0.13, respectively. Non-conversion rates of CpG sites were significantly above the assay background of unconverted cytosines at non-CpG sites in all samples (*p* = 0.0001).

**Figure 8:**
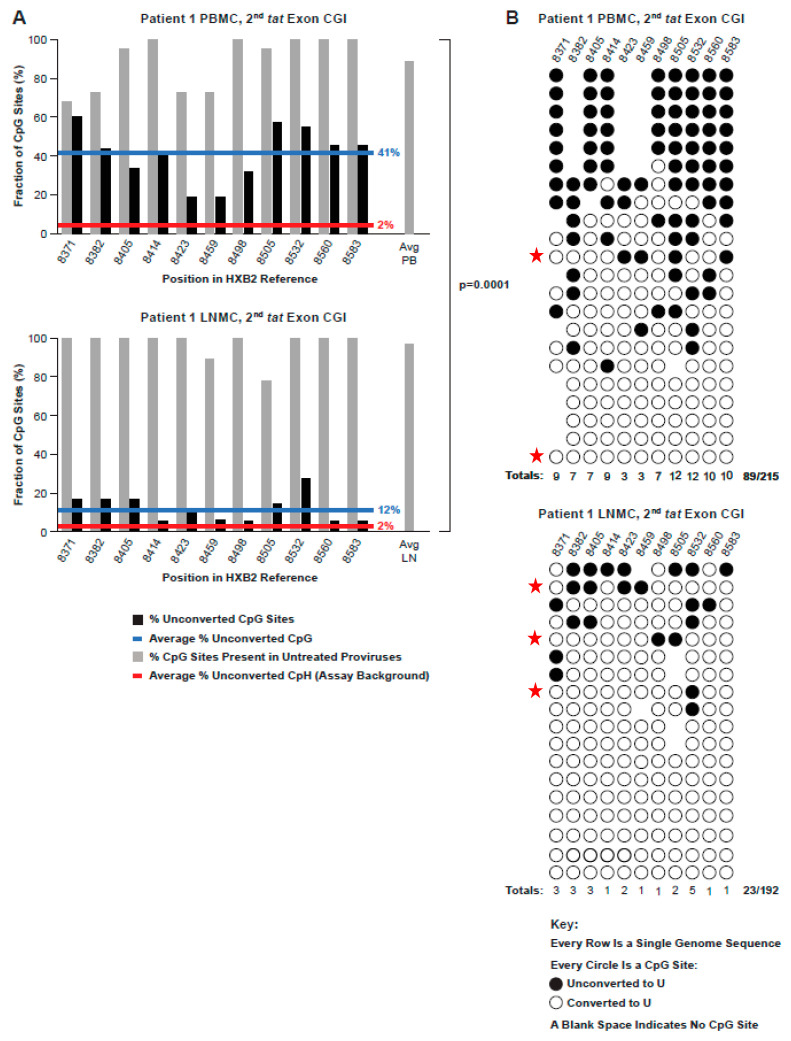
(**A**) Conversion patterns of second *tat* exon CGIs in proviruses extracted from PBMCs and LNMCs from Patient 1. Comparison of the percentage of non-conversion of CpG dinucleotides in the *tat/rev/env* overlap region of Patient 1 in PBMCs (top panel) and LNMCs (bottom). Note that the difference between all PBMCs and LNMCs was significantly different (*p* = 0.0001). (**B**) Conversion profiles of individual CGIs in each single genome from Patient 1 PBMCs and LNMCs. The absence of a dot in a row indicates that the CpG predicted by the consensus sequence was not present in the specific sequence in question. The red stars indicate sequences that are consistent with that of the AMBI-1 clone.

CpG methylation in the HIV-1 CGI located in the overlapping *env-tat-rev* reading frame and encompassing the second exon of *tat* was also analyzed in all donor samples, resulting collectively in 173 bisulfite-converted sequences from PBMCs and LNMCs (Figure 7 and Figure 8). In contrast to 5′ LTR and *gag*-leader CGIs, fractional CpG methylation in this region was consistently above the background (*p* = 0.0001; Supplemental Table S6), though methylation patterns varied considerably among individual proviruses. The average CpG methylation among PBMC and LNMC samples from donors 1683 and 2669 was 22.6%, and ranged from 13.0% (2669 PBMC) to 32.5% (1683 LNMC) (Figure 7). In proviruses from PID 2669, eight of the 13 CpGs were methylated at a frequency of less than 10%, whereas more than 67% of CpGs at position 8474 were methylated in proviruses from the 1683 LNMC sample. However, in these two donors, the fractional methylation detected in the PBMCs was not statistically different from the LNMCs (Figure 7).

Further analysis of proviruses from PID 1683 revealed a large population of identical sequences (albeit the segment sequenced in the second exon of *tat* was very short), where 10/23 of the PBMC and 12/28 of the LN sequences were identical. Despite their identity, these sequences mostly showed different methylation patterns, and there were only two different sets of identical sequences with identical methylation patterns, one set in the LNMC and one in the PBMC set (Supplemental Figure S2).

In contrast, the fractional methylation detected in the PBMCs in PID 1079 was significantly different from the fraction detected in the LNMCs (*p* = 0.0009) (Figure 7). The average methylation level in the second *tat* exon CGI in Patient 1 PBMC-derived proviruses was especially high (41%), and the difference between that measured from the LNMC sample from the same individual (12.0%) was also significant (*p* = 0.0001) (Figure 8A). This variability even extended to the methylation of individual proviruses within samples; for instance, of the 22 Patient 1 PBMC proviruses we evaluated, five were 100% methylated within the second *tat* exon CGI, five were entirely unmethylated, and intermediate methylation frequencies were measured for the remaining sequences (Figure 8B). Among the top five 100% methylated proviruses shown in Figure 8B, all are missing the same three CpG sites at positions 8382, 8423, and 8459. Phylogenic analysis confirmed that two were identical, suggesting the possibility that these proviruses were members of a clonal population (Supplemental Figure S3). This possible clone was different from the AMBI-1 clone. We do not know with certainty which proviruses were AMBI in this region. Nevertheless, we have identified the sequences that are consistent with the signature AMBI sequence in Figure 8B by red stars, which provide yet another example of the considerable heterogeneity in CpG methylation among potentially identical proviruses in the same expanded clone. Although the virological significance of this variation is unclear, we did not find any obvious correlation between second *tat* exon CGI methylation frequencies and the CpG composition of the island.

## 4. Discussion

Identifying and understanding the HIV-1 latent reservoir remains one of the principal challenges in HIV research. Although provirus transcriptional suppression by CpG methylation is well-established in some viruses [12,13,29], and thus seems a likely mechanism for long-term transcriptional suppression of HIV-1, prior studies of provirus methylation in individuals living with HIV have been conflicting. Some reports have described an absence of DNA methylation in the HIV-1 5′ LTR promoter region [16,17], and others have reported significant levels of methylation increasing with prolonged ART [30].

In the present study, we exploit our prior characterization of the AMBI-1 provirus [19] to, for the first time, investigate the 5′ LTR promoter methylation status of a clonal population known to contain a provirus that is both replication-competent and transcriptionally silent [19]. We compared these results with those obtained from a broader sampling of proviruses from Patient 1 and from three other individuals known to have >80% transcriptionally silent proviruses on ART [24]. Collectively, we observed no apparent CpG methylation detectable above the assay background in the 5′ LTR region of AMBI-1 or any other provirus in the samples tested, indicating that DNA methylation of the LTR is not a contributor to HIV-1 transcriptional suppression, even among proviruses known to contribute to the latent reservoir.

Similar results were obtained from a methylation analysis of *gag*-leader CpG islands in proviruses from these individuals, though some minor variation among the samples was observed. Specifically, no apparent CpG methylation above the background was detected in samples from three of the four infected individuals. However, in one donor, there was significant methylation detected across the CGI in the *gag*-leader, albeit at a low level. The general concordance of these data with our analysis of methylation within the 5′ LTR region suggests that the two HIV-1 CpG clusters proximal to the promoter could be considered as parts of a single, functionally integrated CGI. Indeed, this aggregate CGI would more closely resemble CpG islands in the human genome, which typically range from 200–3000 bp in length and often extend well beyond the transcription start site(s) in both the 5′ and 3′ directions [31]. The biological significance of sporadic methylation at three *gag*-leader CpG sites in samples from Patient 1 is unclear, except to demonstrate that methylation patterns can vary among and within infected individuals. Collectively, however, the absence of significant levels of CpG methylation in both the 5′ LTR and *gag*-leader islands supports the conclusion that it is not an important factor in maintaining HIV-1 transcriptional suppression.

In contrast to the results obtained for the 5′ LTR and *gag*-leader, nearly one in four CpG dinucleotides in the second *tat* exon CGI were found to be methylated. Moreover, ours is not an isolated observation, as CpG methylation within the same region in proviruses obtained from clinical samples has been reported previously [16,32]. However, since the methylation patterns were not correlated with the donor or tissue of origin, or the CpG composition of the specific CGI, the cellular or virological determinants for this activity remained unclear. Intragenic CpG methylation is not uncommon in human somatic cells, having been found to accrue with greater frequency than in CpG islands encompassing proximal promoters [10], raising the possibility that there may be previously undescribed effects of CpG methylation on HIV gene expression (such as splicing, for example) [33], rather than on the promoter function. Moreover, the transcription of antisense RNA, which has been previously shown to repress HIV viral gene expression [34], may also contribute to selective intragenic HIV DNA methylation in a manner resembling that observed in the alpha-thalassemia pathology [35].

More likely than a specific functional role, however, is that the higher levels of DNA CpG in the 255-nucleotide second *tat* exon region have been preserved over time because of the three overlapping reading frames, imposing a stringent selection for the specific sequence at this site; mRNAs containing clusters of CpGs are targets for degradation mediated by binding of the ZAP protein [36], and, therefore, CpG dinucleotides are rare in cellular and viral exons. Since CpG sites in DNA are subject to methylation unless actively blocked or reversed [10], the frequent methylation of this site may be irrelevant to provirus expression, and implies that the LTR and *gag*-leader methylation are actively blocked (or reversed), perhaps by (ineffective) transcription factor binding or chromatin modification.

Our finding of predominantly nonmethylated CGIs in or near the HIV-1 promoter region and higher levels of methylation in the second *tat* exon CGI was consistent among the four donors studied in both PBMCs and LNMCs. However, the levels of methylation found in PBMCs verses LNMCs in the second *tat* exon CGI was not consistent. Unlike donors 1683 and 2669, where the average fractional methylation across the tissues was the same, Patient 1 and 1079 had significantly higher levels of methylation in PBMCs than those found in LNMCs. The significance of these results remains unclear; however, it is perhaps related to the differential expansion of some clones. In addition, the distribution of CpG sites in the three CGIs was also not always consistent. For example, in Patient 1 PBMCs, the absence of three CG sites in the second *tat* exon CGI was found in five sequences with identical methylation patterns. This finding could be explained by the expansion of a single infected cell. Apart from this observation, the variability or loss of CpG dinucleotides within a site across the CGI was most evident in the LTR regions. The biological significance is unclear, but it is perhaps best explained by the fact that LTRs are neither translated nor structurally important, and not subject to strong purifying selection as in the *tat* region.

In summary, our data establish that the silencing of HIV proviruses in the cohort studied, particularly of the replication-competent, highly expanded AMBI-1 provirus from Patient 1, cannot be explained by CpG methylation of the 5′ LTR. Therefore, unlike for other retroviruses, DNA methylation of pro-viral LTRs is not the mechanism for HIV latency during ART.

## Data Availability

All sequences are available on the National Cancer Institute HIV Dynamics and Replication Program’s website, found at https://fscigl-trups01p.ncifcrf.gov/index.php.

## Supplementary Materials

The following are available online at https://www.mdpi.com/article/10.3390/v13050799/s1, Figure S1: Neighbor joining trees of HIV-1 5’ LTR SGS from all donors, Figure S2: Conversion profiles of PID 1683, Figure S3: Neighbor joining tree of Patient 1, Table S1: Donor Characteristics, Table S2: (a): SGS primers used in non-bisulfite treated gDNA, (b): Converted SGS primers for bisulfite treated gDNA 5’LTR, (c): Converted SGS primers for bisulfite treated gDNA *gag*-leader, Table S3: SGS of the 5’LTR from Bisulfite treated control proviral DNA, Table S4: SGS of the 5’LTR from bisulfite treated LNMC and PBMC proviral DNA from 4 donors, Table S5: SGS of the *gag*-leader from bisulfite treated LNMC and PBMC proviral DNA from 4 donors, Table S6: SGS of the *2^nd^* exon of *tat* from bisulfite treated LNMC and PBMC proviral DNA from 4 donors.

## Abbreviations

ART	Antiretroviral therapy
CGI	CpG island, (5′ Cytosine—phosphate—Guanine 3′)
CpG	5′ Cytosine—phosphate—Guanine 3′
CpH	5′ Cytosine—phosphate—Adenine, Cytosine or Thymine 3′
dUpH	deoxyuridine—phosphate—Adenine, Cytosine or Thymine
gDNA	Genomic deoxyribonucleic acid
HIV-1	Human Immunodeficiency Virus Type 1
LNMC	Lymph node mononuclear cells
LTR	Long terminal repeat
PBMC	Peripheral blood mononuclear cells
PCR	Polymerase chain reaction
SG	Single genome
SGS	Single genome sequencing
U	Uricil
