# Supplementary material for: CpG Methylation Profiles of HIV-1 Proviral DNA in Individuals on ART
Source: Viruses. 2021 Apr 29;13(5):799. doi: 10.3390/v13050799 (PMC8146454; doi:10.3390/v13050799)
Supplement: Supplementary file 1 [file viruses-13-00799-s001.zip › viruses-1153968 suppl/viruses-1153968 supplementary legend.pdf]

Supplemental Figure 1. Neighbor joining trees of HIV-1 5' LTR SGS obtained from PBMC and LNMC proviral DNA from all participants. Black symbols represent proviral DNA sequences from PBMC and red from LNMC.

Supplemental Figure 2. Conversion profiles of individual CGIs in each single genome from PID 1683 PBMC and LNMC**.** The red stars indicate sequences that are identical. The asterisks indicate the sequences that exhibit both sequence and methylation pattern identity.

Supplemental Figure 3. Neighbor joining tree of the top 6 SGS of bisulfite converted proviral DNA Patient 1 PBMC represented in figure 8B, showing 2 identical SGS.
