# Supplementary material for: CpG Methylation Profiles of HIV-1 Proviral DNA in Individuals on ART
Source: Viruses. 2021 Apr 29;13(5):799. doi: 10.3390/v13050799 (PMC8146454; doi:10.3390/v13050799)
Supplement: Supplementary file 1 [file viruses-13-00799-s001.zip › viruses-1153968 suppl/viruses-1153968 table suppl.pdf]

Supplemental Table 1. Donor Characteristics

Participant identifier (PID)	Sex	Years on ART	Current ART regimen	Plasma RNA copies/ml	Cell type tested	Number of infected cells/million MC [a]	Percent infected cells with unspliced HIV RNA [e]
Patient 1	Male	11	EFV ABC/3TC	<50	PBMC	200	5%
		13	TDF/FTC/RTG	Unknown [b]	LNMC [c]	400	ND [d]
1079	Male	11.4	FTC/TDF EFV	<40	PBMC	137	8%
				<40	LNMC	194	20%
1683	Male	5.4	FTC/TDF RTV, DRV	<40	PBMC	192	5%
				<40	LNMC	154	7%
2669	Male	4.3	ABC/3TC/DTG	<40	PBMC	429	6%
				<40	LNMC	1673	13%

[a] Mononuclear cells
[b] Last documented 6 months prior to time point tested- 107 copies/ml
[c] Autopsy specimen
[d] Not determined
[e] Reference19 and 24

Supplemental Table 2a. SGS primers used in non-bisulfite treated gDNA

**Primer Name**	**Region**	**Sequence**
P-1 55-U3_LTRF	5'LTR- p17 *gag*	ACCACACACAAGGCTACTTCC
P-1 p17-1095R		TCTAAAGCTTCCTTGGTGTCTTTTA
P-2 72-U3F		TTCCCTGATTGGCAGAACTACAC
P-2 p17-1054R		ACACAATAGAGGGTTGCTACTGTATT
P-1 7807F	*env*	AGCAGCAGGAAGCACTATGGGCGC
P-1 8827R		CAAACTACTTTTTGACCACTTGCC
P-2 8217F		TAAATGGGCAAATTTGTGGAATTGG
P-28784R		CCTTTCAAAGCCCTGTCTTATTCTT

Supplemental Table 2b. Converted SGS primers for bisulfite treated gDNA 5'LTR

PID	Primer Name	Sequence 5' to 3'
Patient 1 AMBI-1 clone	P1-UNI5140034FN	TGTTATTAGGGTTAATTTTAGATATTGTGAGGTATGG
	P1-Mut 64R	TTCTCTTTCRCTTTCAAATCCCTATTC
	P2-Mut 38F	GGTGTTTTAAGTTAGTATTAGTTGAGTTTATTG
	P2-Mut 12R	ACACACACTACTTAAAACACTCAAAACAAAC
Patient 1	P1-Mut 9F	AGGGGTTAGATATTTATTGATTTTTGGATGG
	P1-Mut 64R	See above
	P2-Mut 38F	See above
	P2-Mut 12R	See above
1079	P1-69Mut 59F	AGAGGTTAATGAAGGAGAGAATAATAGTTTG
	P1-79Mut 64R	TTTTCCTTTCRCTTTCAAATCCCTATTC
	P2-79Mut 83F	TWGTTTGTTATATTTTATAAGTTAGTATGGAATGGAGG
	P2-Mut 12R	See above
1683	P1-83Mut 59F	AGAGGTTAATAAAGGAGAGAATAATTGTTTG
	P1-83Mut 64R	TTTACTTTCRCTTTCAAATCCCTATTC
	P2-83Mut78F	TWGTTTGTTATATTTTATAAGTTAGTATGGAATGGAGG
	P2-Mut 12R	See above
2669	P1-69Mut 59F	AGAGGTTAATGAAGGAGAGAATAATAGTTTG
	P1-Mut 64R	See above
	P2-69Mut 83F	TAGTTTGTTATATTTTATGAGTTTGTATGGGATGGATG
	P2-Mut 12R	See above

Supplemental Table 2c. Converted SGS primers for bisulfite treated gDNA *gag*-leader

PID	Primer Name	Sequence 5' to 3'
Patient 1	P1-RMut 0F	GTTTTTTTGGTTAGATTAGATTTGAGTTTGGG
	P1-p17Mut 46R	AATCRTTCTAACTCCCTACTTACCCATAC
	P2-RMut 71F	TTAATAAAGTTTGTTTTGAGTGTTTTAAGTAGTG
	P2-p17Mut 83R	TTAATTTATATTTTTTCTTTCCCCCTAACCTTAACC
1079	P1-RMut 0F	See above
	P1-79p17Mut46R	AATCRTTCTAACTCCCTACTTACCCATAC
	P2-RMut 71F	See above
	P2-p17Mut 83R	See above
1683	P1-RMut 0F	See above
	P1-p17Mut 46R	See above
	P2-RMut 71F	See above
	P2-83p17Mut 83R	TTAATTTATATTTTTTCTTTCCCCCTAACCATAACC
2669	P1-RMut 0F	See above
	P1-p17Mut 46R	See above
	P2-RMut 71F	See above
	P2-p17Mut 83R	See above

Supplemental Table 2d. Converted SGS primers for bisulfite treated gDNA *tat* 2nd exon

PID	Primer Name	Sequence 5' to 3'
Patient 1	P1-Mut 0F	TAGATTTAATAAGAATAGAATGAAAAAGAATTGTTGG
	P1-Mut115R	AAACTCTTTATAATACTTCTATAATCCTATCTATCCCC
	P2-Mut 49F	GGGTAAATTTGTGGAATTGGTTTGG
	P2-Mut 128R	TACTTCTATAATCCTATCTATCCCCTCAACTAC
1079	P1-79Mut 8F	AATAGGAAAAGAATGAATAAGAATTATTGTAATTGGA
	P1-79Mut 115R	CAACTCTTTATACTACTTCTATAACCCTATCTATCCCC
	P2-79Mut 42F	AATTGGATTATTGGGTAAGTTTGTGGAATT
	P2-79Mut 118R	CTCTTTATACTACTTCTATAACCCTATCTATCCCCTCA
1683	P1-83Mut 0F	TAGATTTAATAAGATAAGAATGAGTAAGAATTATTGG
	P1-83Mut 115R	AAATTCTTTATATTAATTCTATAACCCTATCTATCCCC
	P2-83Mut 49F	GTGGGTAAGTTTGTGGAATTGGTTTGA
	P2-83Mut 128R	TAATTCTATAACCCTATCTATCCCCTCAACTAC
2669	P1-83Mut 0F	See above
	P1-83Mut 115R	See above
	P2-83Mut 49F	See above
	P2-83Mut 128R	See above

Supplemental Table 3. SGS of the 5'LTR from bisulfite treated Control proviral DNA

Sequence	Site	Number unconverted	Total - sites	Percent	P value
NL4-3 PCR product	CpG	5	224	2.2	0.99
	CpH	32	1288	2.5	
NL4-3 Methyl-free plasmid	CpG	5	160	3.1	0.83
	CpH	44	1100	4.0	
CEM/ACH2 M.SssI	CpG	72	72	100	0.0001
	CpH	2	360	0.6	

Two tailed Fisher's exact test

Supplemental Table 4. SGS of the 5'LTR from bisulfite treated LNMC and PBMC proviral DNA from 4 donors

Sequence	Site	Number unconverted	Total - sites	Percent	P value
5'LTR AMBI-1	CpG	2	90	2.2	0.64
	CpH	6	410	1.5	
5'LTR Aggregate Patient 1	CpG	16	539	3.0	0.99
	CpH	77	2937	2.6	
5'LTR PID 1079	CpG	11	392	2.8	0.86
	CpH	53	2039	2.6	
5'LTR PID 1683	CpG	13	439	3.0	0.40
	CpH	58	2553	2.3	
5'LTR PID 2669	CpG	6	304	2.0	0.69
	CpH	45	1784	2.5	

Two tailed Fisher's exact test

Supplemental Table 5. SGS of the *gag* leader from bisulfite treated LNMC and PBMC proviral DNA from 4 donors

**Sequence**	**Site**	**Number unconverted**	**Total - sites**	**Percent**	**P value**
gag-leader Patient 1	CpG	38	677	5.6	0.0001
	CpH	44	1993	2.2	
gag-leader PID 1079	CpG	13	528	2.5	0.99
	CpH	38	1468	2.6	
gag-leader PID 1683	CpG	12	585	2.1	0.75
	CpH	41	1682	2.4	
gag-leader PID 2669	CpG	18	789	2.3	0.07
	CpH	70	1909	3.7	

Two tailed Fisher's exact test

Supplemental Table 6. SGS of the 2nd exon of *tat* from bisulfite treated LNMC and PBMC proviral DNA from 4 donors

**Sequence**	**Site**	**Number unconverted**	**Total - sites**	**Percent**	**P value**
2nd exon of *tat* Patient 1	CpG	112	407	27.5	0.0001
	CpH	41	2333	1.8	
2nd exon of *tat* PID 1079	CpG	138	590	23.4	0.0001
	CpH	65	2480	2.6	
2nd exon of *tat* PID 1683	CpG	159	570	27.9	0.0001
	CpH	95	3116	3.0	
2nd exon of *tat* PID 2669	CpG	85	550	15.5	0.0001
	CpH	72	2316	3.1	

Two tailed Fisher's exact test
