# Supplementary figures and images for: CpG Methylation Profiles of HIV-1 Proviral DNA in Individuals on ART
Source: Viruses. 2021 Apr 29;13(5):799. doi: 10.3390/v13050799 (PMC8146454; doi:10.3390/v13050799)

# Supplemental Figure 1

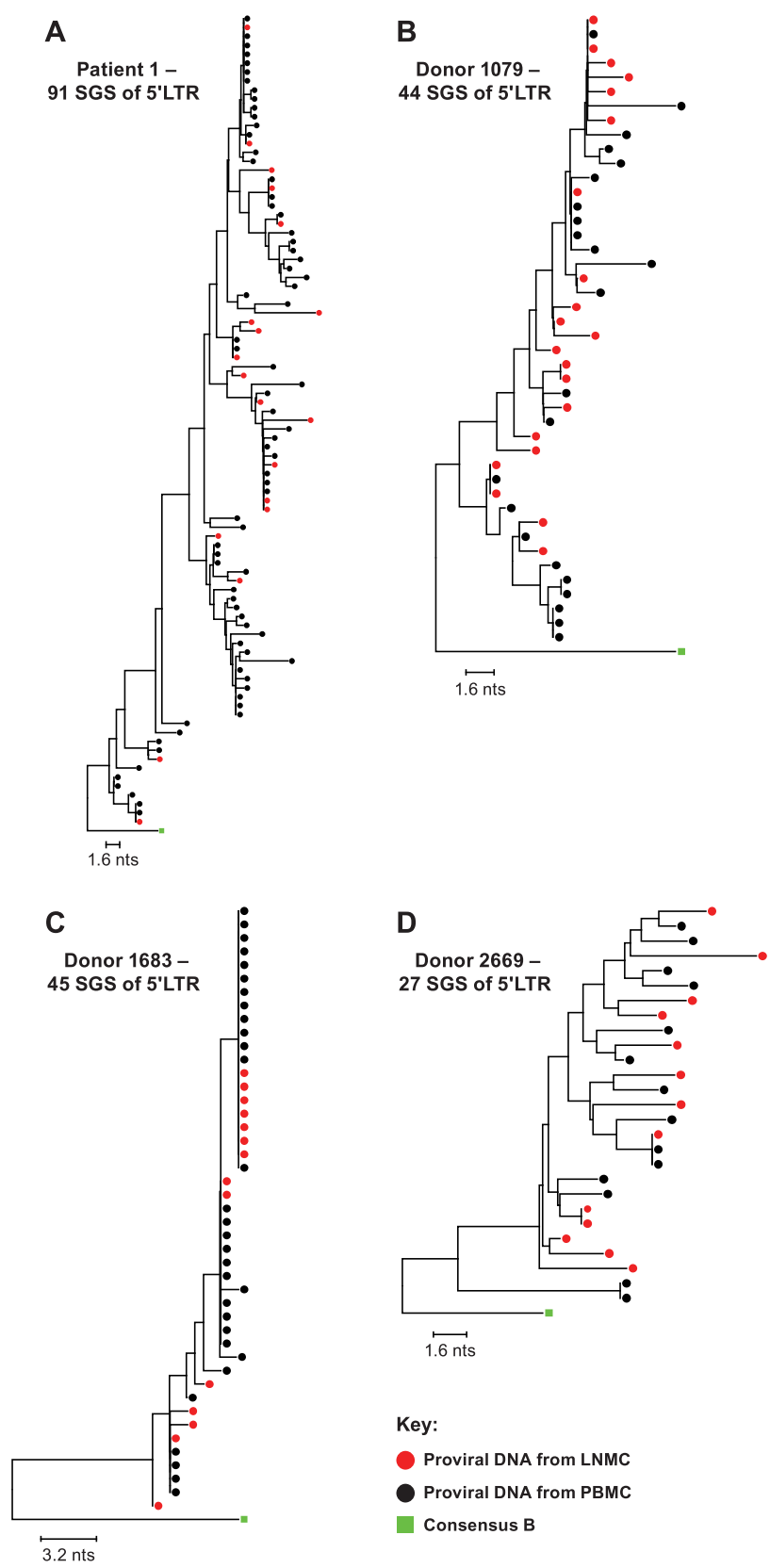

# Supplemental Figure 2

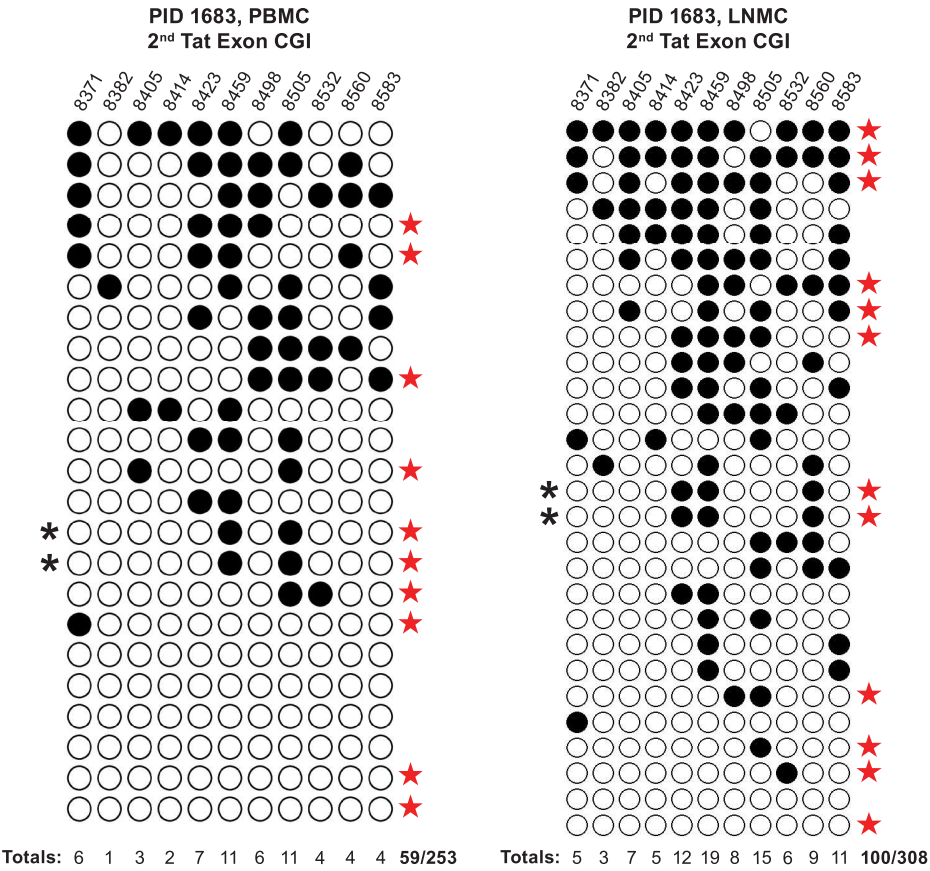

## Supplemental Figure 3

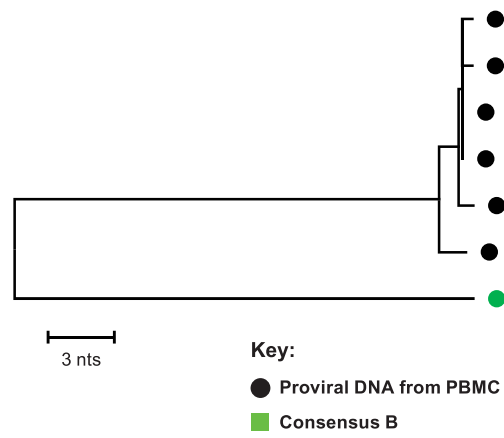

Supplement: Supplementary file 1 [file viruses-13-00799-s001.zip › viruses-1153968 suppl/viruses-1153968 figure suppl.pdf]
